# Which features of postural sway are effective in distinguishing Parkinson's disease from controls? A systematic review

**DOI:** 10.1002/brb3.1929

**Published:** 2020-11-04

**Authors:** Wenbo Ge, Christian J. Lueck, Deborah Apthorp, Hanna Suominen

**Affiliations:** ^1^ Research School of Computer Science Australian National University Canberra ACT Australia; ^2^ Department of Neurology Canberra Hospital Canberra ACT Australia; ^3^ Australian National University Medical School Canberra ACT Australia; ^4^ School of Psychology University of New England Armidale NSW Australia; ^5^ Machine Learning Research Group Data61/CSIRO Canberra ACT Australia; ^6^ Department of Future Technologies University of Turku Turku Finland

**Keywords:** machine learning, meta‐analysis, Parkinson's disease, postural control, systematic review

## Abstract

**Background:**

Postural sway may be useful as an objective measure of Parkinson's disease (PD). Existing studies have analyzed many different features of sway using different experimental paradigms. We aimed to determine what features have been used to measure sway and then to assess which feature(s) best differentiate PD patients from controls. We also aimed to determine whether any refinements might improve discriminative power and so assist in standardizing experimental conditions and analysis of data.

**Methods:**

In this systematic review of the literature, effect size (ES) was calculated for every feature reported by each article and then collapsed across articles where appropriate. The influence of clinical medication status, visual state, and sampling rate on ES was also assessed.

**Results:**

Four hundred and forty‐three papers were retrieved. 25 contained enough information for further analysis. The most commonly used features were not the most effective (e.g., PathLength, used 14 times, had ES of 0.47, while TotalEnergy, used only once, had ES of 1.78). Increased sampling rate was associated with increased ES (PathLength ES increased to 1.12 at 100 Hz from 0.40 at 10 Hz). Measurement during “OFF” clinical status was associated with increased ES (PathLength ES was 0.83 OFF compared to 0.21 ON).

**Conclusions:**

This review identified promising features for analysis of postural sway in PD, recommending a sampling rate of 100 Hz and studying patients when OFF to maximize ES. ES complements statistical significance as it is clinically relevant and is easily compared across experiments. We suggest that machine learning is a promising tool for the future analysis of postural sway in PD.

## Graphical abstract

Existing studies have analyzed many different features of postural sway in an attempt to characterize Parkinson's disease. Here, we perform a systematic literature review and find the effect size of those features collapsed across several articles. The purpose of this was to create a comprehensive list of features that have been adequately explored, what features require more investigation, and what features are yet to be explored at all.

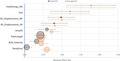

## 1 INTRODUCTION

*Parkinson's disease* (PD) is the second most prevalent neurodegenerative disease (Jankovic, 2008; Kalia & Lang, 2015; Nutt & Wooten, 2005). It evolves slowly over time and has a well‐recognized prodromal period before symptoms and signs become apparent (Gonera et al., 1997; Hawkes et al., 2010). Diagnosis is currently made clinically, based on history, examination, and response to medication (Savitt et al., (2006)). Thus, however skilled the clinician, diagnosis remains somewhat subjective, and this potentially contributes to significant rates of delayed diagnosis and misdiagnosis (Media PA, 2019). The most commonly used tool to measure PD patients' disease status and severity is the Movement Disorders Society's Revision of the *Unified Parkinson's Disease Rating Score* (UPDRS) (Goetz et al., 2008) which suffers from considerable inter‐rater variability (Heldman et al., 2011; Post et al., 2005). The development of a more accurate and quantifiable marker would introduce greater objectivity into the diagnostic process, allow more accurate tracking of disease severity, and facilitate clinical management (Fahn, 2005; Pålhagen et al., 2006; Whone et al., 2003).

Maintenance of posture is a complex process requiring input from visual, vestibular, and somatosensory systems (Winter, 1995). Several methods have been used to assess postural sway, the most common of which uses a force plate to quantify the movement of an individual's *center of pressure* (CoP) while standing. Research suggests that assessment of postural sway might provide an objective, and potentially more accurate, way of assessing PD (Mancini et al., 2012; Souza Fortaleza et al., 2017).

To date, published studies have investigated numerous different features of postural sway recorded under varying experimental conditions. The choice of feature used in any study depends on multiple factors, including the equipment used and, often, preconceptions based on previous experimental results. Because existing studies do not analyze the same features as each other or, indeed, all possible features, it remains unclear which feature(s) and experimental conditions provide maximum discriminative power between PD patients and controls, and hence the most clinically meaningful information. *Machine learning* (ML) is a promising approach that could be used to provide a better idea of which features are the most clinically meaningful, and it has the ability to determine which feature (or set of features) has the largest discriminative power.

As a prelude to the wider use of ML in this field, it is important to survey what information is already available in the literature. Accordingly, the primary aim of this systematic review was to determine what postural sway features have been used to date in the literature and which features appear to be most effective at distinguishing a PD patient from a healthy individual. Classification of patients into the different subtypes of parkinsonism and assessment of disease severity, while important, were considered to be beyond the scope of this review. By limiting ourselves to simply distinguishing patients from controls, we sought to determine what experimental refinements might improve discriminative power when analyzing sway data, and so provide suggestions regarding optimization of data analysis in the future.

## 2 METHODS

The review followed PRISMA guidelines for systematic reviews (Liberati et al., 2009). SCOPUS, Web of Science, PubMed, the Cochrane Library, and the IEEE Xplore Digital Library databases were searched in August 2018 using the following search strategy: “TITLE‐ABS‐KEY(parkinson*) AND TITLE‐ABS‐KEY(stabilo* OR statokine* OR postur* OR sway) AND TITLE‐ABS‐KEY(static)”. Retrieved articles were screened for duplicates and independently assessed for inclusion by two reviewers; conflicts were resolved by a third reviewer.

Inclusion criteria were as follows: First, studies had to include both idiopathic PD group (to avoid confusion we excluded explicit diagnoses of “Parkinson's plus” syndromes) and *healthy control* (HC) groups. Second, studies had to analyze static postural sway using an objective and quantifiable approach (other than clinicians' scores/ratings) and attempt to classify participants as PD or HC based on postural measures. Third, papers had to be available in English as full‐length articles.

For further subanalysis, articles that split the PD group into subgroups such as “fallers” or “nonfallers” were collapsed into one PD group. The effectiveness was only calculated between control and PD groups if the difference in average age was within six years, as age is known to affect postural sway (Røgind et al., 2003). If experimental details of the task were not explicit, the task was assumed to involve static standing with eyes open. We compared articles that reported medians and interquartile ranges with those that reported means and standard deviations. There was no clear difference; thus, all features and their effectiveness were assumed to be normally distributed, allowing conversion of medians, interquartile ranges, and *confidence intervals* (CI) into means and *standard deviations* (*SD*), and *vice versa*. Four studies did not provide adequate numerical information, presenting their results only graphically. For these articles, an attempt was made to contact the authors. If it was not possible to obtain the original numerical values, these were estimated as accurately as possible from the published graphs.

For every article, the discriminatory power of every feature analyzed was represented as an *effect size* (ES), defined as the difference between the means of PD and HC groups divided by the variance of the two groups. ESs were then collapsed across different articles using two separate methods: a *weighted average*, weighted by number of PD participants in each study, and *pooling*, which generates an ES equivalent to that which would be generated if data from every individual participant in all experiments were available, rather than a simple average of summary statistics (Rudmin, 2010) (see Multimedia Appendix S1 for more details). These two methods captured different aspects of the data, such that a large difference in the results would cast doubt on the reliability of a particular feature's ES.

Effect size was also analyzed as a function of the sampling rate used by the force plate, medication/clinical status, and visual status of PD patients (i.e., eyes open or closed). Regarding medication/clinical status, “ON” and “OFF” referred to a patient being less affected by their PD (i.e., more mobile and less tremulous) and at “baseline”/unmedicated parkinsonian state, respectively. A 95% CI was calculated for each ES.

## 3 RESULTS

**FIGURE 1:**
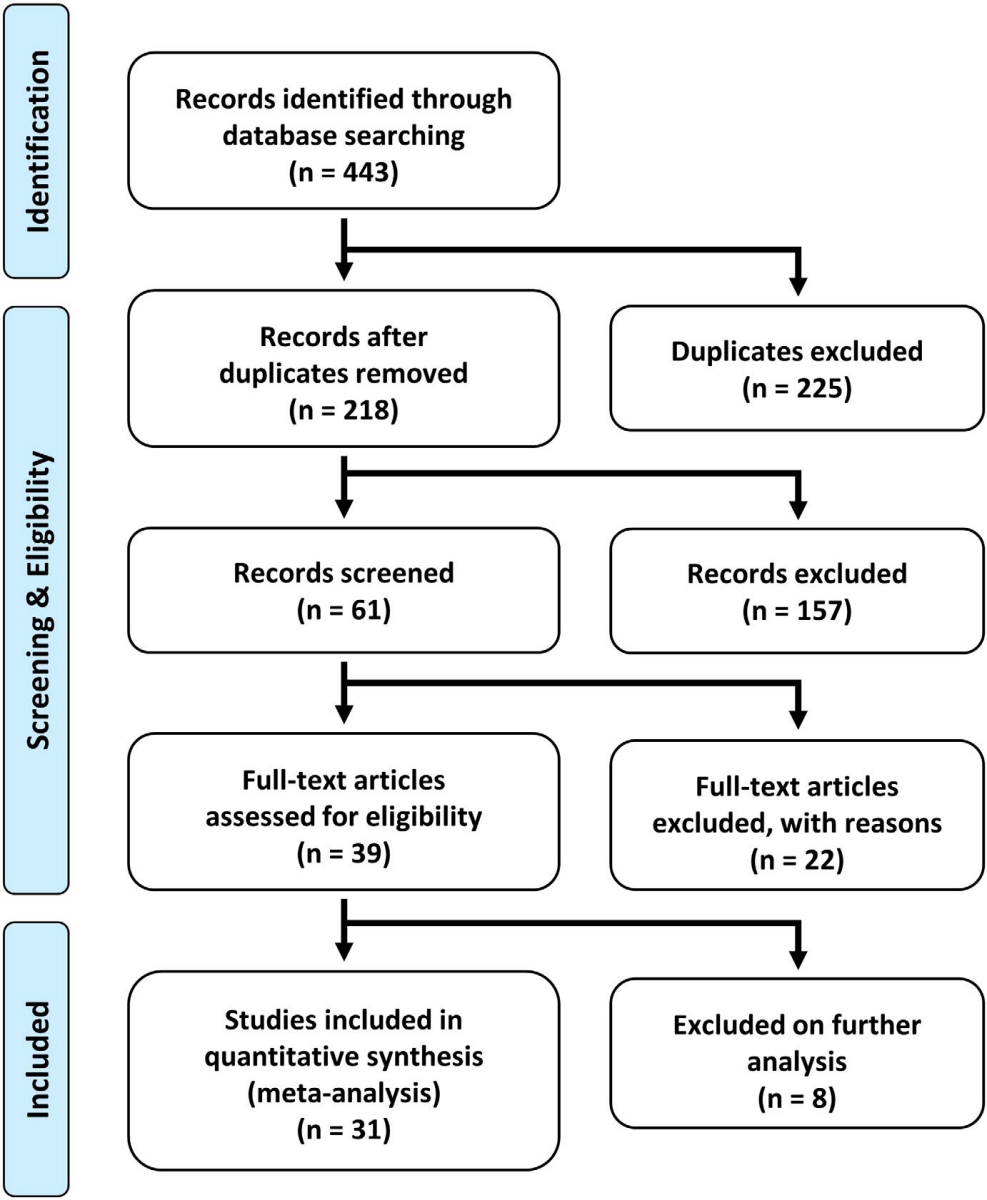
PRISMA flow diagram

As shown in Figure 1, the literature search generated 443 articles, which was reduced to 218 after removing duplicates. This was further reduced to 61 on the basis of the titles and abstracts of the articles. After applying the inclusion/exclusion criteria to the full article, 39 remained, of which 31 included the quantitative information we were looking for (Figure 1). A full list of articles reviewed is provided in Multimedia Appendix S2—Table S1.

### 3.1 Features and effectiveness

**FIGURE 2:**
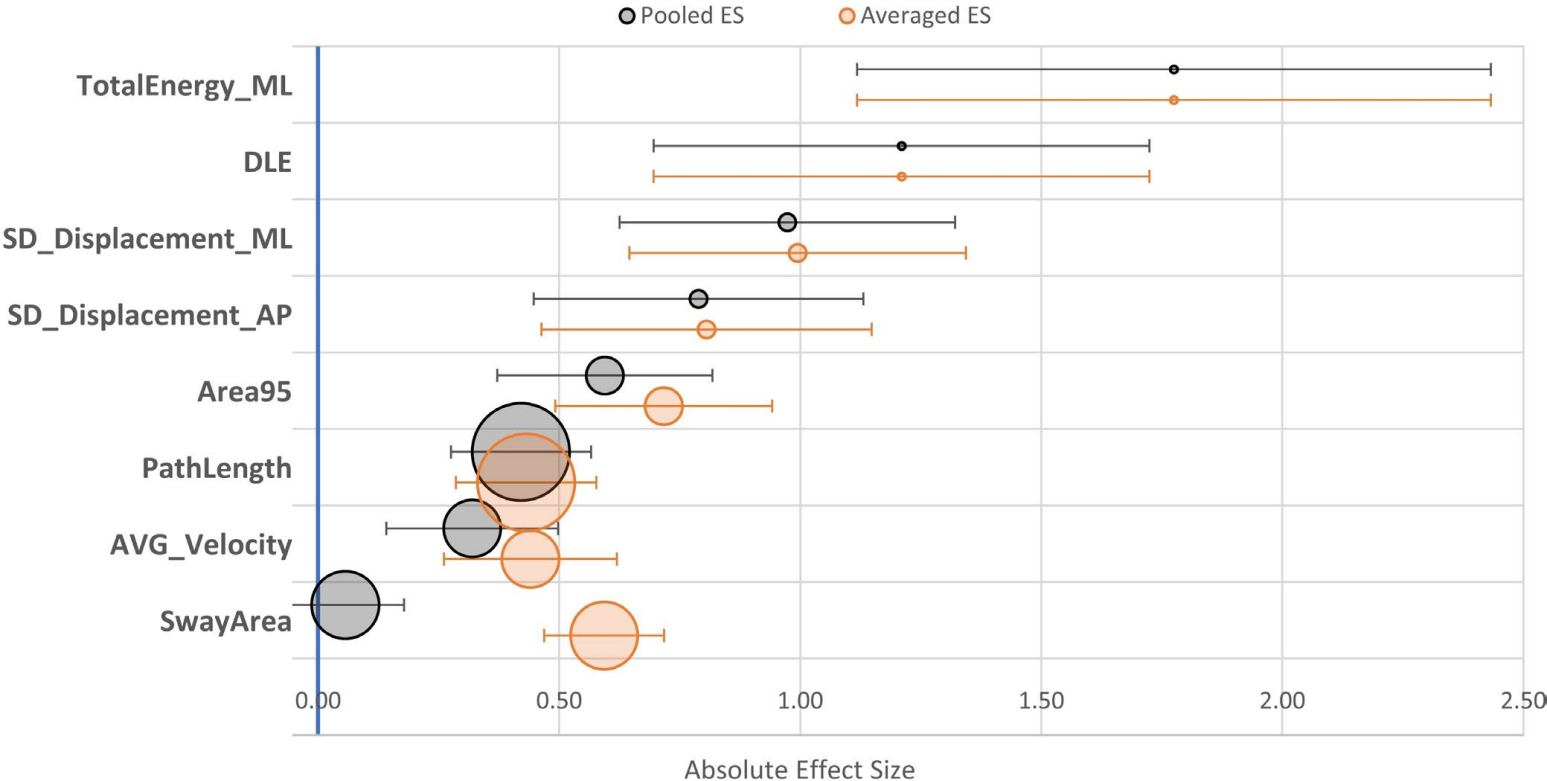
Forest plot of effect sizes of selected feature. The diameter of each circle reflects the number of articles using that feature. Error bars represent 95% confidence intervals. (See Multimedia Appendix S2—Table S2 for definitions and Multimedia Appendix S2—Figure S1 for complete graph.) AP, anteroposterior; AVG, average; ML, mediolateral; *SD*, standard deviation

Overall, 129 different features were used in the 31 studies. The features that were used in at least 4 studies for analysis of postural sway are listed in Table 1. The ES could only be derived for 23 of the 31 studies. These were collapsed across studies where appropriate (Figure 2). The other six studies did not provide adequate information to allow calculation of ES or did not meet the conditions for subanalysis.

**TABLE 1:** Most common features used for analysis of postural sway (further information available in Multimedia Appendix S2—Table S2)

Feature name	Number of studies using feature
PathLength	14
Area95	9
SwayArea	9
AVG_Velocity	9
AVG_Veloctiy_AP	5
PathLength_AP	5
PathLength_ML	5
AVG_Velocity_ML	4
RMS_Dispalcement_AP	4
RMS_Dispalcement_ML	4
SD_Displacement_AP	4
SD_Displacement_ML	4

Abbreviations: AP, anteroposterior; AVG, average; ML, mediolateral; RMS, root‐mean‐square; *SD*, standard deviation.

The most commonly used features, for example, PathLength or SwayArea, did not appear to be the most effective. Total energy in the mediolateral direction had the largest ES but was only used in one article (27 participants), and this is reflected in its wide confidence intervals. *SD* of displacement in anteroposterior and mediolateral directions were used in two articles (total of 79 participants) with medium‐to‐large ES (see Multimedia Appendix S2—Figure S1 and Table S3 for complete graph and numerical values).

### 3.2 Experimental conditions

**FIGURE 3:**
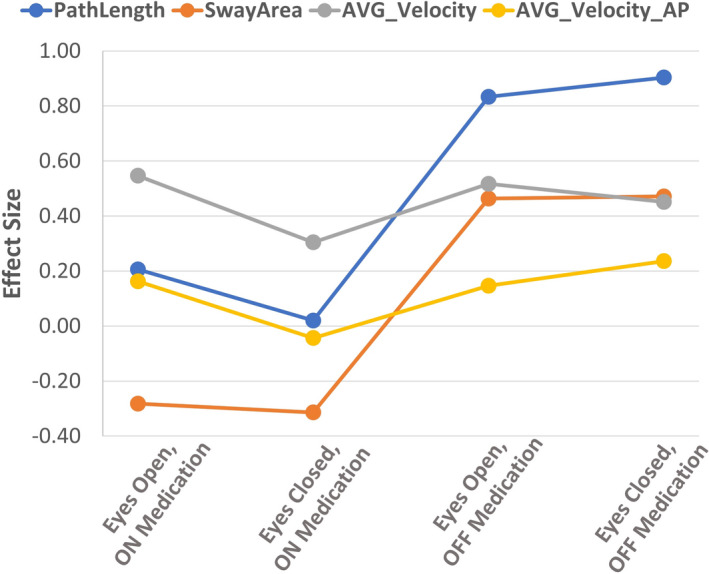
Effect sizes of features common to all experimental conditions and used in at least two articles. [Complete data in Multimedia Appendix S2—Table S4]. AP, anteroposterior; AVG, average

Figure 3 shows the effect of clinical status (ON or OFF) and visual state, that is, *eyes open* (EO) or *eyes closed* (EC) on ES. PathLength and SwayArea demonstrated larger ES when the patient was OFF compared to ON but there was no clear effect of visual status.

**FIGURE 4:**
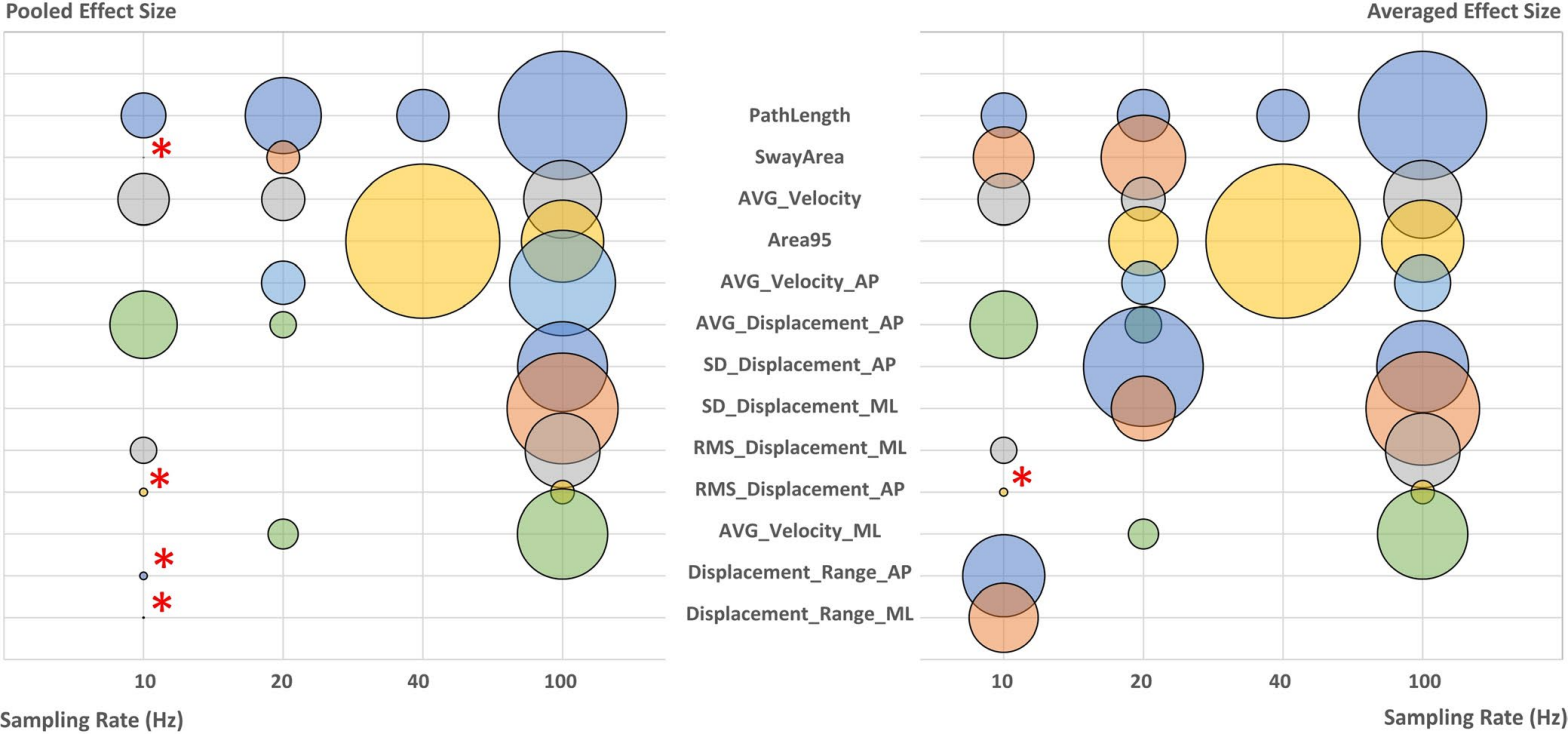
Pooled (left) and averaged (right) effect sizes (represented as circle area) of features as a function of sampling rate. Very small effect sizes have been marked with a red asterisk. AP, anteroposterior; AVG, average; ML, mediolateral; RMS, root‐mean‐square; *SD*, standard deviation

Of the 23 papers which allowed for subanalysis, 14 reported the sampling rate of the force plate used. A low sampling rate resulted in a smaller ES (Figure 4). Note that in Figure 4, ESs of several features are represented as very small circles, for example, pooled ES for SwayArea, RMS of displacement in the AP direction, and displacement range in both AP and ML directions at 10 Hz, and averaged ES for RMS of displacement in the AP direction at 10 Hz. These have been marked with a red asterisk for visibility.

### 3.3 Statistical tests

Every article reviewed used a test of statistical significance to assess performance of the various features. However, the choice of test varied greatly between articles, partly affected by sample size, test conditions, assumption of normality, and, possibly, familiarity with statistics. Only two articles used a *receiver operating characteristic* (ROC) curve, and none presented a confusion matrix or metrics such as accuracy, specificity, or sensitivity.

## 4 DISCUSSION

The purpose of this review was to begin to move toward more objective and quantifiable testing of PD. To the best of our knowledge, there has been no previous comprehensive analysis of the various features of postural sway used in the investigation of PD. Our results show that the most commonly used features, such as PathLength or Area95, may not be the most effective in terms of discriminative power, while there were other promising features that require further investigation. Studying patients when OFF appears to increase the ES of certain features, but there was no consistent effect of vision. For any given feature, increased sampling rate was associated with an increase in ES. Finally, there was enormous variability in the statistical tests used by the various authors.

### 4.1 Features and effectiveness

Though ES is a good representation of a feature's discriminative ability, the 95% CI can be a bit misleading as they do not take into account how many experiments contributed to the overall result. Larger numbers of experiments are likely to reduce the effects of bias more than larger numbers of subjects in a single experiment since the single study may have been subject to bias. With multiple independent experiments, there is a smaller chance of the same persistent bias. This nuance is not reflected in the CI. Accordingly, if an ES was derived from fewer than three independent articles, it should be considered as relatively less reliable.

Of the features that we have confidence in, none has a large ES, indicating that current methods probably would not work well on their own in discriminating PD patients from controls and that further research is needed to find more clinically useful measures. Of the features that we are not confident in, ones that have a large ES should be investigated further, such as total energy in the mediolateral direction, dynamic Lyapunov exponent, and standard deviation of displacement in the mediolateral direction. We think investigation of certain features, such as ones derived from *recurrence quantification analysis* (RQA) and diffusion plot analysis, requires added caution as the parameters of these can be configured in such a way that they result in a large ES for the data in the study, but the findings may not generalize well beyond the sample data. Such methods require additional performance evaluation, such as cross‐validation on an unseen dataset (Kohavi, 1995). It is also important to point out that there is a large number of possible features that have not yet been investigated (Christ et al., 2018), any one of which may effectively capture the difference in sway between a PD patient and a healthy individual.

### 4.2 Experimental conditions

In general, being OFF increased ES, but this was not seen for all features. The reason is unclear: It may simply represent overall insensitivity of a particular feature, but alternatively, it may be that some features are independent of disease status. Granted that an effect of disease statues on SwayArea and PathLength was observed across several studies involving many participants, it is likely that the effect is real.

There was no consistent effect of vision status. Some authors have suggested that vision helps to stabilize posture in PD (Bronstein et al., 1990; Frenklach et al., 2009; Louie et al., 2009; Panyakaew et al., 2015), while others disagree (Cattaneo et al., 2016; Paolucci et al., 2018, Schmit et al., 2006). One possible explanation of these contradictory results is that the effects of vision might vary with disease severity: Like healthy individuals, less severely affected patients might rely on visual input while more severe patients do not (Paolucci et al., 2018). As above, this study was not designed to look at the effects of disease severity so it is not possible to make further comment on this.

The ES was influenced by sampling rate. The sampling rate of the force plate is known to affect the numerical value of certain features (Raymakers et al., 2005). However, this does not explain why a lower sampling rate is associated with a smaller ES. While chance is always a possible explanation, it is possible that lower sampling rates reflect lower‐quality equipment which might be more susceptible to noise. Alternatively, it could also be that a lower sampling rate really does decrease effectiveness. The Nyquist–Shannon sampling theorem states that if a function contains no frequency higher than *B* Hz, it can be completely determined by sampling at 2*B* Hz (the Nyquist rate) (Shannon, 1949). Accordingly, a sampling rate of approximately 10 Hz should be adequate since parkinsonian tremor occurs at 3–6 Hz (Baumann, 2012) and postural sway occurs at frequencies below 5 Hz (Loram et al., 2006). However, as sampling rate decreases and approaches the Nyquist rate, a given signal needs to be sampled for a longer time period, particularly with nonperiodic signals such as postural sway. Shorter time periods result in imperfect signal reconstruction, an effect, which can be avoided by using a sampling rate much higher than the Nyquist rate.

### 4.3 Statistical analysis

All studies used some form of test to determine whether a given feature differed significantly between groups. However, statistical significance is not the same as clinical meaningfulness or usefulness: Significance simply indicates how likely the results are, or are not, to be due to chance, and is heavily influenced by the number of participants (Sullivan & Feinn, 2012). An ES, on the other hand, gives a better indication of whether a feature is capable of meaningfully differentiating between individuals, as it takes account of both the magnitude of the mean difference between groups and the overall variance of the feature in question (i.e., it is a measure of signal‐to‐noise ratio).

Of course, the *p* value is important, but including the ES renders results more meaningful and more easily comparable (Ialongo, 2016). It is easy to calculate the ES, and we therefore suggest that this measure should be provided in future studies. Other metrics that demonstrate the true discriminative power of a feature include a confusion matrix (when testing on an unseen data set) or a receiver operator characteristic (ROC), and these should also be considered.

### 4.4 Call for standardization and objectivity

There was a clear lack of standardization of both the experimental setup (such as stance width, medication status, device used, and tasks performed) and the statistical testing, meaning that it is difficult to make reliable comparisons between studies. This heterogeneity may be one of the key reasons why objective testing methods in PD are not yet clinically useful. In an attempt to reduce heterogeneity between studies, thereby facilitating the discovery of clinically useful features, we offer a few recommendations when assessing sway during quiet, static standing (Table 2).

**TABLE 2:** Recommendations for data acquisition when assessing postural sway

	Recommended
Sampling rate	100 Hz
Medication state	OFF^a^
Visual state	Record both eyes open and eyes closed conditions (but the difference may not be meaningful, particularly in patients with more severe disease)
Performance metric	In addition to statistical significance testing, provide effect size, confusion matrix, and ROC where relevant

^a^ At least 12 hr after last dose of antiparkinsonian medication (Frenklach et al., 2009).

### 4.5 Machine learning

Machine learning is an established tool that can discriminate between groups by learning an optimal set of parameters for a given model. This review of the literature suggests that it is well suited to the analysis of postural sway in PD for several reasons. First, it has the potential to be more objective than the current methods used in diagnosis. Second, ML methods can utilize complex nonlinear interactions between many features to increase discriminative power. Third, the standard performance metrics applied to ML are more easily compared across studies. Importantly, a trained model that performs well in distinguishing participants with PD from HC is likely to be a strong contender as an objective measure of severity.

However, ML has potential drawbacks. Some modern and powerful algorithms, such as neural network‐based deep learning models, require a very large amount of annotated (training) data, something which is rarely available from medical studies. Also, these models may be too abstract, making them difficult to apply in clinical practice. Simpler ML models, such as *support vector machines* (SVM) or random forests, do not require as much training data but can still create robust models. One example is the well‐studied handwritten digit recognition task (Lecun et al., 1998; LeCun,n.d.); the best performing SVM has a classification error or only 0.56% (Decoste & Schölkopf, 2002), while the best deep network has a classification error of 0.23% (Cireşan et al., 2012). Also, the use of transfer learning may enable the use of deep learning in the medical field where data is limited (Zhou et al., 2019).

### 4.6 Limitations

Publication bias may mean that the real ESs of some features are smaller than the values reported here, especially for those features that have been used less frequently. The small group sizes of some articles mean it is not possible to test normality. Nevertheless, we have treated the features as normally distributed; otherwise, it would not be possible to draw any meaningful conclusions, since it was necessary to assume normality when calculating ES. These kinds of assumptions are inherent to meta‐analysis and could be tested in future studies.

Four articles did not report numerical values of means and SDs, but instead presented their results graphically. Wherever possible, these authors were contacted in order to obtain their original data. If the authors could not be contacted, values were estimated directly from graphs, so some values may have been subject to interpretation error.

It is possible that any of the studies may have included patients with atypical PD, such as progressive supranuclear palsy or multiple system atrophy, rather than idiopathic PD. This may have increased heterogeneity and reduced ES. However, it is often very difficult to distinguish these patients in the early stages of their disease and this problem therefore applies to all studies of postural sway. It is hoped that finding a more objective measure of Parkinson's disease will make it possible to distinguish these conditions more accurately in the future.

## 5 CONCLUSION

Objective and quantifiable PD classification is not yet possible, and much work is still required. However, this systematic review has revealed many important points. The most commonly used features for analyzing postural sway are unlikely to be the most effective, and there are many other features which have not yet been adequately explored. We have also identified relationships between the ES of a feature and certain experimental conditions such as clinical state and sampling rate of the force plate.

One possible reason why objective diagnostic tools for PD are still not available relates to the heterogeneity of experimental details, analysis tools, and methods of reporting used in different studies. We have recommended experimental conditions that are likely to increase the effectiveness of certain features in discriminating PD patients from healthy controls, along with performance metrics that are better able to demonstrate clinical importance and discriminative ability and, in turn, allow comparison between studies. We hope this review will assist in guiding future research.

## CONFLICT OF INTERESTS

Nothing to declare.

## AUTHOR CONTRIBUTION

Wenbo Ge contributed to all aspects of this work. Christian Lueck contributed significantly to the conceptualization and writing of this work. Deborah Apthorp contributed significantly to the analysis and with valuable resources. Hanna Suominen contributed significantly to data curation and the supervision of this work. Additionally, all three (Christian, Deborah, and Hanna) provided invaluable contributions in the conceptualization, supervision, reviewing, and editing of this work.

### Peer Review

The peer review history for this article is available at https://publons.com/publon/10.1002/brb3.1929.

## Supporting information

Appendix S1

Appendix S2

## Data Availability

The data that support the findings of this study are all from the literature and can be found online. The specific articles are listed in Appendix S2—Table S1. Additionally, data generated from the analysis of the literature are available from: https://osf.io/v53rg/?view_only=c6cbbfb9b4614e4188a8d434897492b4
