# Supplementary material for: Which features of postural sway are effective in distinguishing Parkinson's disease from controls? A systematic review
Source: Brain Behav. 2020 Nov 4;11(1):e01929. doi: 10.1002/brb3.1929 (PMC7821610; doi:10.1002/brb3.1929)
Supplement: Supplementary file 1 — Appendix S1 [file BRB3-11-e01929-s001.pdf]

Multimedia Appendix 1: Calculating and combining effect sizes

Various different effect sizes exist, here we use Hedge's g in favour of Cohen's d or Glass' Δ as it captures differences in sample size and variance better.[1] Calculated as:

$$g = \frac{\overline{X_{PD}} - \overline{X_{HC}}}{SD^*}, \qquad SD^* = \sqrt{\frac{(n_{PD} - 1)(SD_{PD}^2) + (n_{HC} - 1)(SD_{HC}^2)}{n_{PD} + n_{HC} - 2}}$$

where n refers to number of participants in a group, SD refers to standard deviation of a group, and $\bar{X}$ refers to average of a feature for a group. Subscripts denote the group, PD being Parkinson's disease group, HC being healthy control group.

This effect size was calculated for every feature used within the reviewed literature, and combined across different articles through two methods: averaging and pooling.

For the **averaging method**, effect sizes were combined with a weighted average, weighting by number of PD participants:

$$g_{averaged} = \frac{\sum g_i \, n_{i,PD}}{\sum n_{i,PD}}$$

summing over i, all articles that contain a certain feature.

For the **pooling method**, effect sizes across studies were combined by first calculating the pooled mean and pooled standard deviation for both the PD and HC groups with the equations below:[2]

$$\overline{X_{pooled}} = \frac{\sum n_i \overline{X_i}}{\sum n_i}, \qquad \mathrm{SD}_{pooled} = \sqrt{\frac{\sum \left[(n_i - 1)SD_i^2 + n_i\left(\overline{X_i} - \overline{X_{pooled}}\right)^2\right]}{\sum[n_i] - 1}}$$

summing over i, all articles that contain a certain feature. The combined effect size is then calculated as:

$$g_{pooled} = \frac{\overline{X_{PD,pooled}} - \overline{X_{HC,pooled}}}{SD_{pooled}^*},$$

where

$$SD_{pooled}^* = \sqrt{\frac{\left(n_{PD,pooled} - 1\right)\left(SD_{PD,pooled}^2\right) + \left(n_{HC,pooled} - 1\right)\left(SD_{HC,pooled}^2\right)}{n_{PD,pooled} + n_{HC,pooled} - 2}}.$$

Additionally, a bias correction is applied if $N < 50$, where N is the combined participant size ($N = n_{PD} + n_{HC}$).[3] For the averaging method, this N refers to the participant size in each individual article, i.e., applied to the effect sizes before average. For the pooling method, this N refers to the participant size in total for a feature, i.e., applied after pooling.

$$g_{unbiased} = g\left(1 - \frac{3}{4N - 9}\right).$$

In the cases where means were replaced with medians, and standard deviations were replaced with inter-quartile ranges (IQR), or 95% confidence intervals ($CI_{95\%}$), normality was assumed, and the means and standard deviations were derived as follows:[4,5]

$$mean = median, \qquad SD = \frac{IQR}{1.35} = \sqrt{N}\left(\frac{CI_{upper,95\%} - CI_{lower,95\%}}{2 * 1.96}\right).$$

A 95% confidence interval for the effect size of each feature is also derived by assuming normality of effect sizes, calculated as:[6]

$$CI_{95\%} = g \pm 1.96\hat{\sigma}\,, \qquad \hat{\sigma} = \sqrt{\frac{n_{PD} + n_{HC}}{n_{PD}n_{HC}} + \frac{g^2}{2(n_{PD} + n_{HC})}}$$

1 Grissom RJ, Kim JJ. *Effect sizes for research: A broad practical approach*. Mahwah, NJ, US: : Lawrence Erlbaum Associates Publishers 2005.

2 Rudmin JW. Calculating the Exact Pooled Variance. *arXiv:10071012 [physics]* Published Online First: 6 July 2010.http://arxiv.org/abs/1007.1012 (accessed 10 Dec 2018).

3 Hedges LV, Olkin I. CHAPTER 14 - Estimation of Effect Size When Not All Study Outcomes are Observed. In: Hedges LV, Olkin I, eds. *Statistical Methods for Meta-Analysis*. San Diego: : Academic Press 1985. 285–309. doi:10.1016/B978-0-08-057065-5.50019-1

4 Wan X, Wang W, Liu J, *et al.* Estimating the sample mean and standard deviation from the sample size, median, range and/or interquartile range. *BMC Med Res Methodol* 2014;**14**:135. doi:10.1186/1471-2288-14-135

5 Higgins JP, Deeks JJ. Chapter 7: Selecting studies and collecting data. In: *Cochrane Handbook for Systematic Reviews of Interventions*.www.handbook.cochrane.org

6 Hedges LV, Olkin I. CHAPTER 5 - Estimation of a Single Effect Size: Parametric and Nonparametric Methods. In: Hedges LV, Olkin I, eds. *Statistical Methods for Meta-Analysis*. San Diego: : Academic Press 1985. 75–106. doi:10.1016/B978-0-08-057065-5.50010-5
