# Supplementary material for: Which features of postural sway are effective in distinguishing Parkinson's disease from controls? A systematic review
Source: Brain Behav. 2020 Nov 4;11(1):e01929. doi: 10.1002/brb3.1929 (PMC7821610; doi:10.1002/brb3.1929)
Supplement: Supplementary file 2 — Appendix S2 [file BRB3-11-e01929-s002.pdf]

Multimedia Appendix 1

Table 1: Studies examined in the systematic literature review.

Author	Year	Full Reference
Paolucci et al.	2018	Paolucci T, Iosa M, Morone G, *et al.* Romberg ratio coefficient in quiet stance and postural control in Parkinson's disease. *Neurol Sci* 2018;:1–6. doi:10.1007/s10072-018-3423-1
De la Casa-Fages et al.	2017	Casa-Fages BD la, Alonso-Frech F, Grandas F. Effect of subthalamic nucleus deep brain stimulation on balance in Parkinson's disease: A static posturographic analysis. *Gait & Posture* 2017;**52**:374–80. doi:10.1016/j.gaitpost.2016.12.025
Blaszczyk	2016	Błaszczyk JW. The use of force-plate posturography in the assessment of postural instability. *Gait & Posture* 2016;**44**:1–6. doi:10.1016/j.gaitpost.2015.10.014
Cattaneo et al.	2016	Cattaneo D, Carpinella I, Aprile I, *et al.* Comparison of upright balance in stroke, Parkinson and multiple sclerosis. *Acta Neurologica Scandinavica* 2016;**133**:346–54. doi:10.1111/ane.12466
Barbosa et al.	2015	Barbosa AF, Souza C de O, Chen J, *et al.* The competition with a concurrent cognitive task affects posturographic measures in patients with Parkinson disease. *Arquivos de Neuro-Psiquiatria* 2015;**73**:906–12. doi:10.1590/0004-282X20150153
Beretta et al.	2015	Beretta VS, Gobbi LTB, Lirani-Silva E, *et al.* Challenging postural tasks increase asymmetry in patients with Parkinson's disease. *PLOS ONE* 2015;**10**:e0137722. doi:10.1371/journal.pone.0137722
Geroin et al.	2015	Geroin C, Smania N, Schena F, *et al.* Does the Pisa syndrome affect postural control, balance, and gait in patients with Parkinson's disease? An observational cross-sectional study. *Parkinsonism & Related Disorders* 2015;**21**:736–41. doi:10.1016/j.parkreldis.2015.04.020
Johnson et al.	2015	Johnson L, Rodrigues J, Teo W-P, *et al.* Interactive effects of GPI stimulation and levodopa on postural control in Parkinson's disease. *Gait & Posture* 2015;**41**:929–34. doi:10.1016/j.gaitpost.2015.03.346
Panyakaew et al.	2015	Panyakaew P, Anan C, Bhidayasiri R. Visual deprivation elicits subclinical postural inflexibilities in early Parkinson's disease. *Journal of the Neurological Sciences* 2015;**349**:214–9. doi:10.1016/j.jns.2015.01.022
Nantel & Bronte-Stewart	2014	Nantel J, Bronte-Stewart H. The effect of medication and the role of postural instability in different components of freezing of gait (FOG). *Parkinsonism & Related Disorders* 2014;**20**:447–51. doi:10.1016/j.parkreldis.2014.01.017
Zawadka-Kunikowska et al.	2014	Zawadka-Kunikowska M, Zalewski P, Klawe JJ, *et al.* Age-related changes in cognitive function and postural control in Parkinson's disease. *Aging Clin Exp Res* 2014;**26**:505–10. doi:10.1007/s40520-014-0209-z
Johnson et al.	2013	Johnson L, James I, Rodrigues J, *et al.* Clinical and posturographic correlates of falling in Parkinson's disease. *Movement Disorders* 2013;**28**:1250–6. doi:10.1002/mds.25449
Vervoot et al.	2013	Vervoort G, Nackaerts E, Mohammadi F, *et al.* Which aspects of postural control differentiate between patients with Parkinson's disease with and without freezing of gait? Parkinson's Disease. 2013. doi:10.1155/2013/971480
Ickenstein et al.	2012	Ickenstein GW, Ambach H, Kloeditz A, *et al.* Static posturography in aging and Parkinson's disease. *Front Aging Neurosci* 2012;**4**. doi:10.3389/fnagi.2012.00020
Colnat-Coulbois et al.	2011	Colnat-Coulbois S, Gauchard GC, Maillard L, *et al.* Management of postural sensory conflict and dynamic balance control in late-stage Parkinson's disease. *Neuroscience* 2011;**193**:363–9. doi:10.1016/j.neuroscience.2011.04.043
Ebersbach & Gunkel	2011	Ebersbach G, Gunkel M. Posturography reflects clinical imbalance in Parkinson's disease. *Movement Disorders* 2011;**26**:241–6. doi:10.1002/mds.23189
Suarez et al.	2011	Suarez H, Geisinger D, Ferreira ED, *et al.* Balance in Parkinson's disease patients changing the visual input. *Brazilian Journal of Otorhinolaryngology* 2011;**77**:651–5. doi:10.1590/S1808-86942011000500019
Benninger et al.	2010	Benninger DH, Michel J, Waldvogel D, *et al.* REM sleep behavior disorder is not linked to postural instability and gait dysfunction in Parkinson. *Movement Disorders* 2010;**25**:1597–604. doi:10.1002/mds.23121
Frenklach et al.	2009	Frenklach A, Louie S, Koop MM, *et al.* Excessive postural sway and the risk of falls at different stages of Parkinson's disease. *Movement Disorders* 2009;**24**:377–85. doi:10.1002/mds.22358
Chastan et al.	2008	Chastan N, Debono B, Maltête D, *et al.* Discordance between measured postural instability and absence of clinical symptoms in Parkinson's disease patients in the early stages of the disease. *Movement Disorders* 2008;**23**:366–72. doi:10.1002/mds.21840

Schmit et al.	2006	Schmit JM, Riley MA, Dalvi A, *et al.* Deterministic center of pressure patterns characterize postural instability in Parkinson's disease. *Exp Brain Res* 2006;**168**:357–67. doi:10.1007/s00221-005-0094-y
Raymakers et al.	2005	Raymakers JA, Samson MM, Verhaar HJJ. The assessment of body sway and the choice of the stability parameter(s). *Gait & Posture* 2005;**21**:48–58. doi:10.1016/j.gaitpost.2003.11.006
Fioretti et al.	2004	Fioretti S, Guidi M, Ladislao L, *et al.* Analysis and reliability of posturographic parameters in Parkinson patients at an early stage. In: Dumont G, Liang Z-P, Hudson DL, eds. *The 26th Annual International Conference of the IEEE Engineering in Medicine and Biology Society* . Piscataway, N.J.: : IEEE 2004. 651–4. doi:10.1109/IEMBS.2004.1403242
Hagiwara et al.	2004	Hagiwara N, Hashimoto T, Ikeda S-I. Static balance impairment and its change after pallidotomy in Parkinson's disease. *Movement Disorders* 2004;**19**:437–45. doi:10.1002/mds.10666
Nallegowda et al.	2004	Nallegowda M, Singh U, Handa G, *et al.* Role of sensory input and muscle strength in maintenance of balance, gait, and posture in Parkinson's disease: a pilot study. *American Journal of Physical Medicine & Rehabilitation* 2004;**83**:898. doi:10.1097/01.PHM.0000146505.18244.43
Baratto et al.	2002	Baratto L, Morasso PG, Re C, *et al.* A new look at posturographic analysis in the clinical context: sway-density versus other parameterization techniques. *Motor Control* 2002;**6**:246–70. doi:10.1123/mcj.6.3.246
Rocchi et al.	2002	Rocchi L, Chiari L, Horak FB. Effects of deep brain stimulation and levodopa on postural sway in Parkinson's disease. *Journal of Neurology, Neurosurgery & Psychiatry* 2002;**73**:267–74. doi:10.1136/jnnp.73.3.267
Manabe et al.	2001	Manabe Y, Honda E, Shiro Y, *et al.* Fractal dimension analysis of static stabilometry in Parkinson's disease and spinocerebellar ataxia. *Neurological Research* 2001;**23**:397–404. doi:10.1179/016164101101198613
Contin et al.	1996	Contin M, Riva R, Baruzzi A, *et al.* Postural stability in Parkinson's disease: the effects of disease severity and acute levodopa dosing. *Parkinsonism & Related Disorders* 1996;**2**:29–33. doi:10.1016/1353-8020(95)00008-9
Trenkwalder et al.	1995	Trenkwalder C, Paulus W, Krafczyk S, *et al.* Postural stability differentiates "lower body" from idiopathic parkinsonism. *Acta Neurologica Scandinavica* 1995;**91**:444–52. doi:10.1111/j.1600-0404.1995.tb00444.x
Schieppati & Nardone	1991	Schieppati M, Nardone A. Free and supported stance in Parkinson's disease. The effect of posture and 'postural set' on leg muscle responses to perturbation, and its relation to the severity of the disease. *Brain* 1991;**114**:1227–44. doi:10.1093/brain/114.3.1227

Table 2: Definition of feature families.

Family and Sub-family	Description
Stabilometric Analysis	Any feature typically used in the analysis of CoP, derived from the stabilogram
Area	Features that require the calculation of an area from the stabilogram
Displacement	Features that require the calculation of displacement from the stabilogram
Stability	Features that are derived from the concept of a limit of stability
Stabilometric Other	Features that could not be grouped with the other subfamilies, within Stabilometric Analysis
Velocity	Features that require the calculation of a velocity from the stabilogram
Spectral Analysis	Any feature that is derived from the Fourier transform of the stabilogram, i.e. derived from its spectral content
Band Power	Features derived from determining the amount of power within a frequency band
Frequency Power	Features dervied from calculating the frequency below which a percentage of the total power of the signal is contained
Spectral Other	Features that could not be grouped with the other subfamilies, within Spectral Analysis
Other	Anything that could not be classified into the other families
Diffusion Plot Analysis	Features derived from transforming the stabilogram to a diffusion stabilogram
Incomparable	Features that cannot be compared to each other, i.e. features of the group rather than the individual
Other other	Features that could not be grouped with the other subfamilies, within Other
Polar Transformation	Features dervied from a transformation into a polar coordinate system
Undefined	Features that were not defined
Nonlinear Dynamical Analysis	Any feature that analyzes the postural sway as if it was a nonlinear dynamical system, trying to assess determinism/predictability
Complexity	Features derived by assuming a complex system, characterising something with many parts which interact to result in a higher order emergence
Nonlinear Other	Features that could not be grouped with the other subfamilies, within Nonlinear Dynamical Analysis
RQA	Features dervied from a Recurrance Quantification Analysis, quantifying the number and duration of recurrances of a dynamical system

Table 3: Effect size and 95% confidence intervals of features used.
A full description of the features can be found in Table 2 of this Multimedia Appendix.[b]

Feature Name	ES Pooled	ES Avged	Difference	# PD	# HC	# Articles	+- 95% CI Pooled	+- 95% CI Avged
TotalEnergy_ML	1.7752	1.7752	0.00	9	18	1	0.6574	0.6574
AVG_Velocity_SI_ML	1.6979	1.6979	0.00	20	20	1	0.7229	0.7229
RMS_Displacement_SI_AP	-1.5925	-1.5925	0.00	20	20	1	0.7113	0.7113
AVG_Velocity_SI_AP	1.4639	1.4639	0.00	20	20	1	0.6979	0.6979
DLE	1.2103	1.2103	0.00	10	15	1	0.5141	0.5141
Area95_SI	1.1553	1.1553	0.00	20	20	1	0.6695	0.6695
Area95_MinorAxisLength	1.0204	1.0204	0.00	48	17	1	0.5803	0.5803
RQAEntropy_AP	0.9864	0.9864	0.00	6	6	1	1.1985	1.1985
Maxline_AP	0.9851	0.9851	0.00	6	6	1	1.1983	1.1983
SDT	-0.9820	-0.9820	0.00	21	38	1	0.5616	0.5616
SD_Displacement_ML	0.9732	0.9945	-0.02	46	33	2	0.3481	0.3489
TotalEnergy_AP	0.9496	0.9496	0.00	9	18	1	0.5935	0.5935
%Determinism_AP	0.8834	0.8834	0.00	6	6	1	1.1855	1.1855
%Recurrance_AP	0.8761	0.8761	0.00	6	6	1	1.1846	1.1846
MSCD	0.8516	0.8516	0.00	21	38	1	0.5547	0.5547
DirectionIndex_AP	-0.8025	-0.8025	0.00	54	54	1	0.2772	0.2772
SD_Displacement_AP	0.7891	0.8054	-0.02	46	33	2	0.3419	0.3424
DirectionIndex_ML	0.7820	0.7820	0.00	54	54	1	0.2767	0.2767
SwayMovement_AP	0.7749	0.7749	0.00	28	17	1	0.4075	0.4075
Frequency95_AP	-0.7686	-0.8074	0.04	10	15	1	0.4902	0.4919
RMS_Displacement	0.7589	0.7589	0.00	6	11	1	0.7355	0.7355
SwayMovement_ML	0.7423	0.7423	0.00	28	17	1	0.4066	0.4066
SwayVectorLength	0.6846	0.6846	0.00	54	54	1	0.2744	0.2744
Frequency95	0.6595	0.6595	0.00	6	11	1	0.7302	0.7302
SwayVectorAngle	-0.6006	-0.6006	0.00	54	54	1	0.2727	0.2727
Area95	0.5947	0.7169	-0.12	107	71	4	0.2229	0.2248
EquilibriumScore	-0.5926	-0.6003	0.01	121	35	2	0.2700	0.2701
Frequency95_SI	0.5781	0.5781	0.00	6	11	1	0.7264	0.7264
FractalDimension	0.5690	0.5690	0.00	15	15	1	0.5162	0.5162
PhasePlaneParameter	0.5227	0.5227	0.00	21	38	1	0.5412	0.5412
CI	-0.5195	-0.5195	0.00	21	38	1	0.5411	0.5411
AVG_Displacement_ML	0.4518	0.4518	0.00	30	30	1	0.3624	0.3624
SwayRatio_ML	0.4367	0.4367	0.00	54	54	1	0.2699	0.2699
PathLength	0.4208	0.4314	-0.01	255	175	10	0.1455	0.1456
AVG_Displacement_AP	-0.3930	-0.1841	-0.21	61	55	1	0.2602	0.2582
PeakDisplacement_ML	0.3917	0.3917	0.00	30	30	1	0.3613	0.3613
SwayArea_VRI	0.3871	0.3871	0.00	31	25	1	0.5317	0.5317
AVG_Displacement	-0.3826	-0.4135	0.03	10	15	1	0.4776	0.4783
Area95_MinorAxisTangent	-0.3517	-0.3517	0.00	48	17	1	0.5565	0.5565
SwayRatio_AP	0.3425	0.3425	0.00	54	54	1	0.2687	0.2687
SwayArea_RR	-0.3211	-0.3211	0.00	56	34	1	0.2475	0.2475
AVG_Velocity	0.3195	0.4402	-0.12	122	129	6	0.1783	0.1793
PathLength_ML	-0.2901	0.1032	-0.39	70	62	4	0.2672	0.2660

RMS_Displacement_SI	0.2766	0.2766	0.00	6	11	1	0.7166	0.7166
AVG_Velocity_SI	0.2668	0.2668	0.00	6	11	1	0.7164	0.7164
PathLength_AP	-0.2639	0.1496	-0.41	70	62	4	0.2669	0.2661
RMS_Displacement_ML	-0.2177	0.2020	-0.42	61	39	2	0.3199	0.3198
PeakDisplacement_AP	0.2095	0.2095	0.00	30	30	1	0.3588	0.3588
DC	-0.2058	-0.2058	0.00	21	38	1	0.5342	0.5342
AVG_Velocity_ML	0.1599	0.4845	-0.32	95	68	4	0.2360	0.2390
Area95_MajorAxisLength	0.1373	0.1373	0.00	48	17	1	0.5537	0.5537
Covariance	0.1151	0.1151	0.00	48	17	1	0.5535	0.5535
PathLength_RR	-0.1144	-0.1144	0.00	30	30	1	0.5065	0.5065
RMS_Displacement_AP	-0.0831	0.0635	-0.15	61	39	2	0.3192	0.3191
Displacement_Range_AP	0.0761	0.7218	-0.65	77	72	2	0.1633	0.1682
SurfaceLengthRatio	-0.0719	-0.0719	0.00	9	18	1	0.5660	0.5660
AVG_Velocity_AP	0.0718	0.4111	-0.34	105	83	5	0.2110	0.2131
SwayArea	0.0564	0.5932	-0.54	201	168	7	0.1220	0.1246
PathLength_SI	-0.0356	-0.0356	0.00	20	20	1	0.6199	0.6199
RMS_Displacement_SI_ML	0.0283	0.0283	0.00	20	20	1	0.6198	0.6198
Displacement_Range_ML	-0.0229	0.6093	-0.63	77	72	2	0.1632	0.1668

Figure 1: Effect size and 95% confidence intervals of features used.

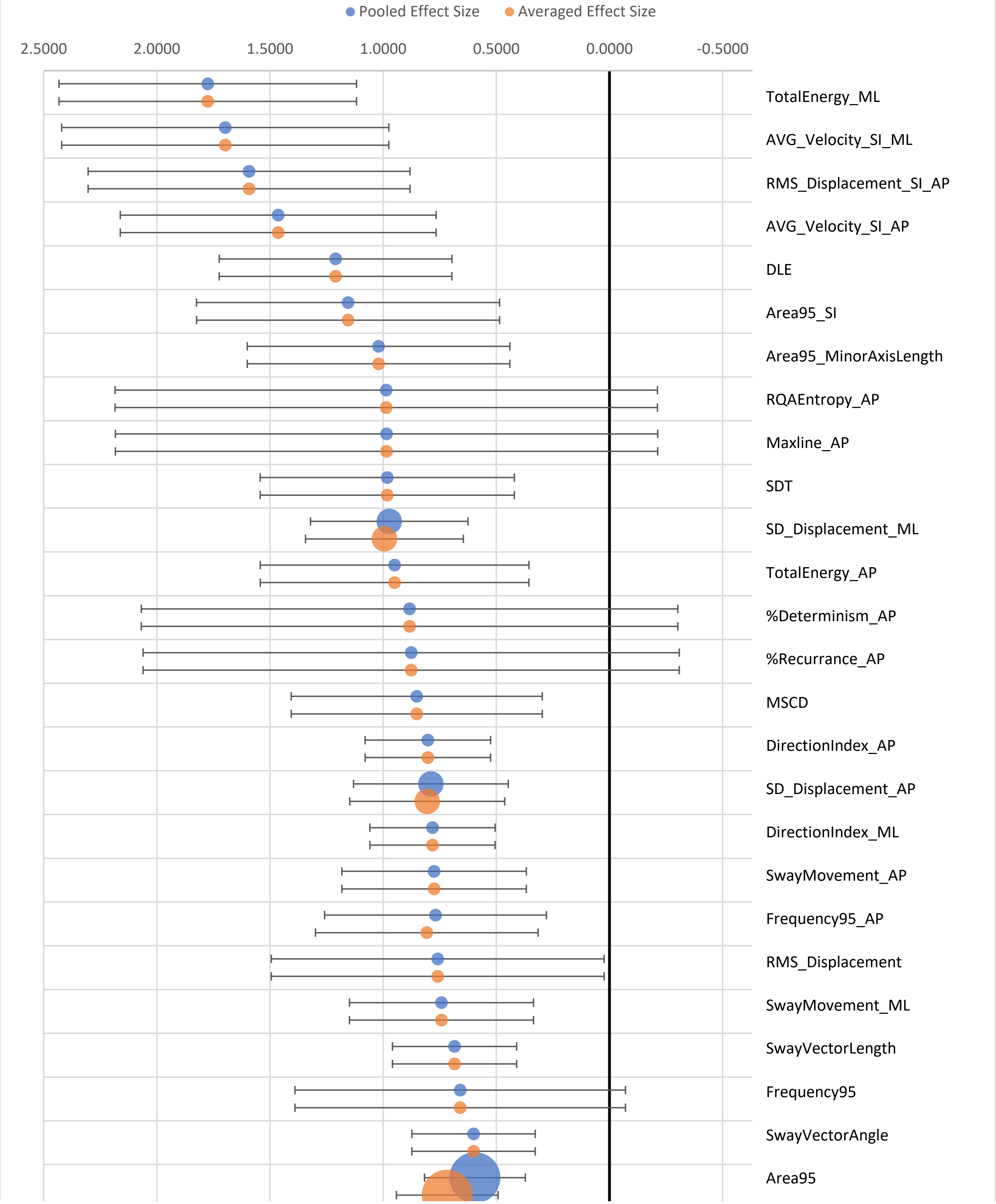

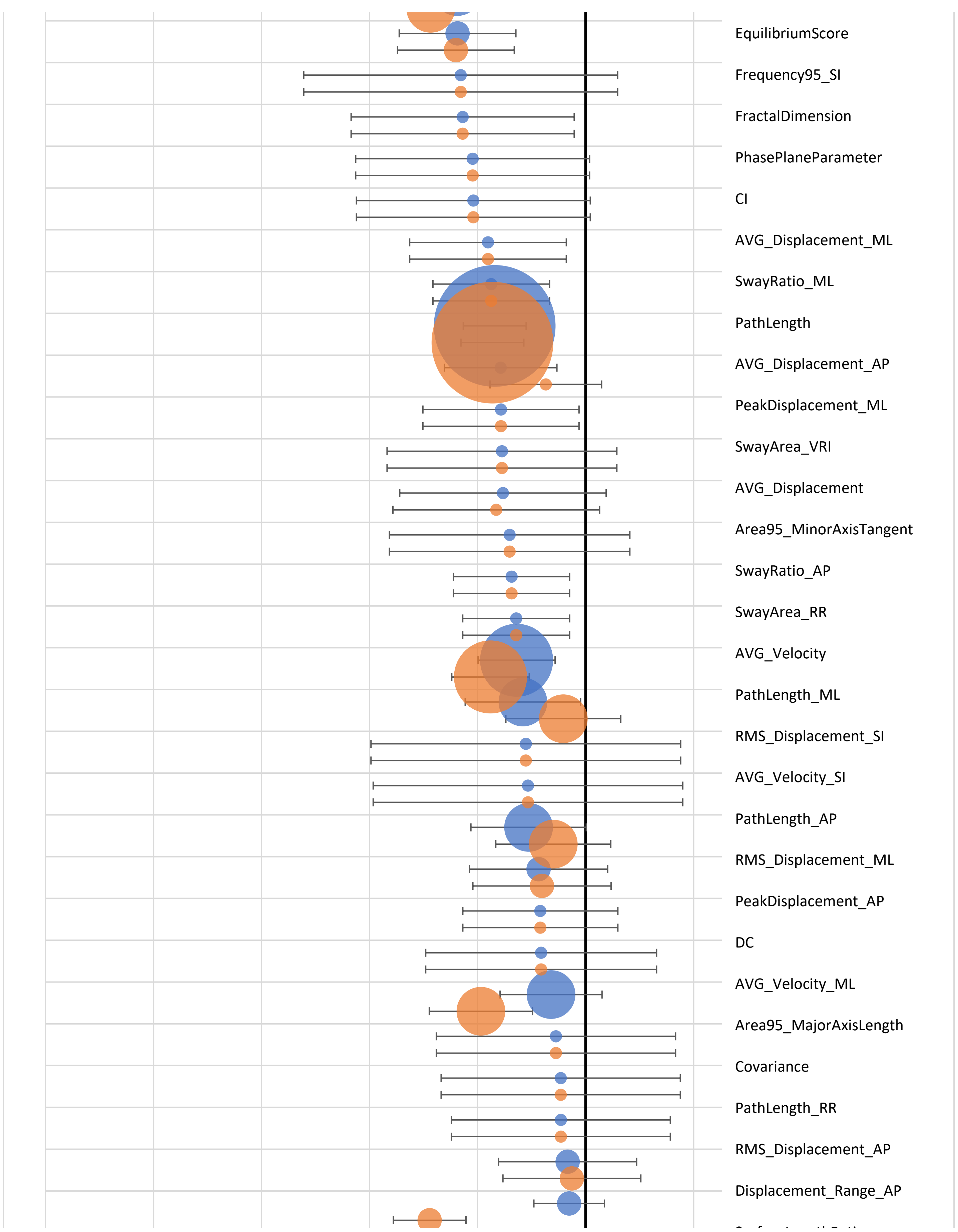

EquilibriumScore
Frequency95_SI
FractalDimension
PhasePlaneParameter
CI
AVG_Displacement_ML
SwayRatio_ML
PathLength
AVG_Displacement_AP
PeakDisplacement_ML
SwayArea_VRI
AVG_Displacement
Area95_MinorAxisTangent
SwayRatio_AP
SwayArea_RR
AVG_Velocity
PathLength_ML
RMS_Displacement_SI
AVG_Velocity_SI
PathLength_AP
RMS_Displacement_ML
PeakDisplacement_AP
DC
AVG_Velocity_ML
Area95_MajorAxisLength
Covariance
PathLength_RR
RMS_Displacement_AP
Displacement_Range_AP

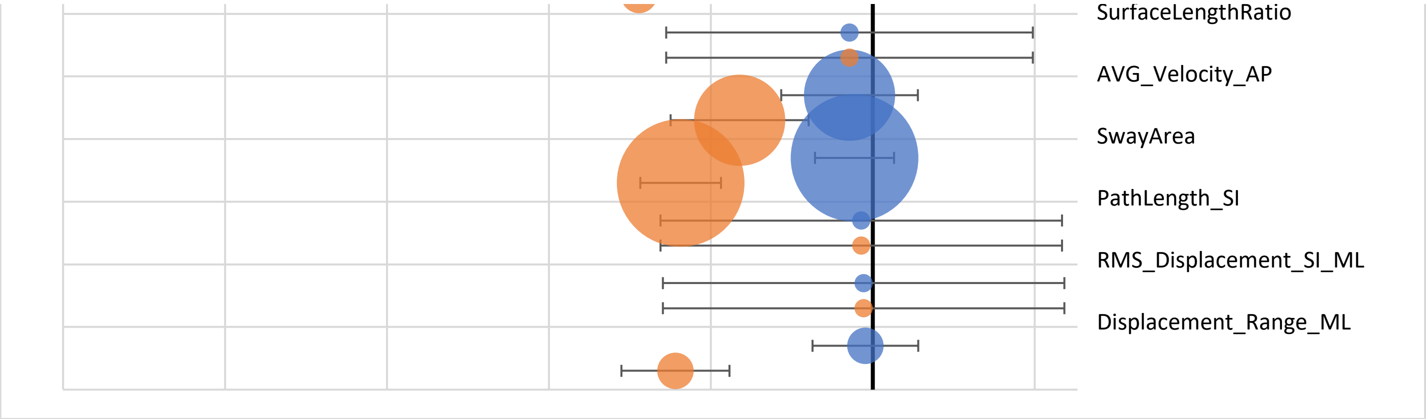

[b]Acronyms and abbreviations:

ES: Effect Size
95% CI: 95% Confidence Interval
RR: Romberg Ratio
SI: Symmetric Index
VRI: Visual Reliance Index
AP: Anteroposterior
ML: Mediolateral
RMS: Root Mean Squared
SD: Standard Deviation
AVG: Average
LOS: Limits of Stability
BFR: Balance Functional Reserve
SDT: Sum of maximal Deviation Time
MSCD: Mean Squared Critical Displacement
DC: Diffusion Constant
CI: Critical Interval
SOTFSS: Sensory Organisation Test Fall Severity Scale
DLE: Dominant Lyapunov Exponent

Table 4: Effect size of features, categorised by vision and medication state.
A full description of the features can be found in Table 2 of this Multimedia Appendix.[c]

	Eyes open, ON medication			Eyes open, OFF medication			Eyes closed, ON medication			Eyes closed, OFFmedication		
**Feature name**	**ES**	**m**	**95% CI**	**ES**	**m**	**95% CI**	**ES**	**m**	**95% CI**	**ES**	**m**	**95% CI**
PathLength	0.21	5	0.2371	0.83	3	0.2874	0.02	3	0.3652	0.90	3	0.3214
SwayArea	-0.28	3	0.2209	0.46	3	0.2305	-0.31	3	0.2212	0.47	3	0.2306
AVG_Velocity	0.55	4	0.3160	0.52	3	0.3598	0.30	3	0.3292	0.45	2	0.3838
AVG_Velocity_AP	0.16	3	0.3634	0.15	3	0.3185	-0.04	3	0.3756	0.24	2	0.3908
AVG_Velocity_ML	0.44	2	0.4122	-0.09	2	0.3473	0.21	2	0.4264	0.38	1	0.4499
Area95	0.59	3	0.3407	0.89	1	0.6932	0.76	2	0.4432	0.78	1	0.7183
AVG_Displacement	-0.23	1	0.8027	-0.35	1	0.8059	-0.28	1	0.8039	-0.67	1	0.8214
EquilibriumScore	-0.70	1	0.5738	-0.67	1	0.3920	-0.76	1	0.5766	-0.72	1	0.3934
Displacement_Range_AP	0.82	1	0.2792	0.54	1	0.3314	0.78	1	0.2782	0.59	1	0.3324
Displacement_Range_ML	0.82	1	0.2793	0.73	1	0.3358	0.59	1	0.2740	0.20	1	0.3267
Frequency95_AP	-2.00	1	0.9728	0.00	1	0.8002	-1.09	1	0.8556	-0.64	1	0.8199
SwayArea_RR	-0.30	1	0.2698	-0.38	1	0.3287	-0.30	1	0.2698	-0.38	1	0.3287
DLE	0.97	1	0.8439	1.67	1	0.9238	1.58	1	0.9128	1.38	1	0.8873

[c]Acronyms and abbreviations:

ES: Effect size
m: number of articles
95% CI: 95% Confidence Interval
AVG: Average
AP: Anteroposterior
ML: Mediolateral
RR: Romberg Ratio
DLE: Dominant Lyapunov Exponent
